# Interpretation of the Genotype by Tissue Interactions of Four Genes (AFP1, CIRP, YB-1, and HMGB1) in *Takifugu rubripes* Under Different Low-Temperature Conditions

**DOI:** 10.3389/fmolb.2022.897935

**Published:** 2022-06-30

**Authors:** Xinan Wang, Zhifeng Liu, Aijun Ma

**Affiliations:** ^1^ Yellow Sea Fisheries Research Institute, Chinese Academy of Fishery Sciences, Qingdao, China; ^2^ Laboratory for Marine Biology and Biotechnology, Qingdao National Laboratory for Marine Science and Technology, Qingdao, China

**Keywords:** genotype, tissue, interactions, Takifugu rubripes, resistance genes to low temperature

## Abstract

**Background:** The differential expression of the same gene in different tissues could be due to the genotype effect, tissue effect, and/or genotype × tissue interactions. However, the genetic mechanisms responsible for this differential expression have not been reported to date.

**Methods:** Four resistance genes to low temperature, the genes for antifreeze protein (AFP), cold induced RNA binding protein (CIRP), high mobility group protein box-1 (HMGB1), and Y-box binding protein (YB-1), were measured by PCR in the liver, spleen, kidney, brain, heart, intestine, muscle, gonad, and skin of *Takifugu rubripes* cultured under different temperature conditions (18, 13, 8, and 5°C). Split-split-plot analysis of variance, additive main effects and multiplicative interaction (AMMI) and genotype main effects and genotype × environment interaction (GGE) biplot analysis were used to evaluate the effects of genotype × tissue interactions on gene expression.

**Results:** The results of split-split-plot analysis of variance showed that water temperature has a significant effect on the expression of *T. rubripes* cold resistant genes, while tissue × gene interaction has a highly significant effect on it. AMMI analysis showed that the contributions of genotype, tissue, and genotype × tissue interactions to the total variation in gene expression followed two trends: 1) as temperature decreased, the gene effect increased gradually and the genotype × tissue interaction decreased gradually; 2) the gene effect at 18 and 13°C was significantly lower than that at 8 and 5°C, while the interaction at 18 and 13°C was significantly higher than that at 8 and 5°C. GGE analysis showed that: at all temperatures except 8°C, the expression rankings of all four genes were highly positively correlated in all tissues except muscle; the expression stability of the genes was the same at 18°C/13°C and at 8°C/5°C; and AFP1 showed the best expression and stability among the four genes.

**Conclusion:** 8°C/5°C as the suitable temperature for such experiments for *T. rubripes*. Among the four antifreeze genes evaluated in this study, AFP1 had the best expression and stability.

## 1 Introduction

Water temperature plays an important role in the growth and reproduction of aquatic animals. Water temperature that is too low leads to weakened fish activity and decreased levels of neurohormone secretion and digestive enzyme activity, resulting in the reduction of growth performance and even death (Abdel-Tawwab and Wafeek, 2014; Wen et al., 2017; Islam et al., 2019; Islam et al., 2020). The suitable growth temperature of the warm-water fish *Takifugu rubripes* is 16–25 °C, and colder water temperatures in winter seriously affect its growth performance. At present, heating the water of indoor tanks is usually used to solve this problem, but it is expensive and not environmentally friendly. Therefore, studying the molecular mechanisms involved in cold adaptation of *T. rubripes* and cultivating cold-resistant varieties is an alternative approach.

Studies of the mechanisms of cold tolerance of fish have been increasing since the 1960s. With the continuous development of molecular biology techniques, numerous genes related to cold tolerance have been discovered and applied to practical production. Antifreeze proteins (AFPs) were first found in polar marine organisms, and later studies reported that they are widespread in fish, plants, insects, bacteria, and fungi (Duman and Olsen, 1993; Cui et al., 2019; Xu et al., 2019; Zhang et al., 2020). AFPs are adsorbed onto the surface of ice crystals, where they inhibit the growth rate of ice crystals and reduce the freezing point of water molecules, thereby conferring cold resistance (Raymond and DeVries, 1977; Wang et al., 2012). Numerous studies of AFPs in fish have also been conducted (Hew et al., 1999; Zhong and Fan, 2002; Cai L. Y. et al., 2018). Early studies reported that cold-induced RNA binding protein (CIRP) was closely related to hypothermia stimulation, and its expression increased significantly with decreasing temperature (Nishiyama et al., 1997). In a low temperature environment, CIRP slows down the rate of apoptosis by inhibiting cell division and reducing the demand for nutrients, and it coordinates the transcription and translation of multiple genes (Al-Fageeh and Smales, 2006). In recent years, researchers have found that CIRP is involved in the response to low temperature stress in some fish (Hu et al., 2015; Miao et al., 2017; Xu et al., 2018). High speed migration protein family protein (HMGB1), which is ubiquitous in eukaryotes and highly conservative, participates in DNA transcription, recombination, and repair (Lange and Vasquez, 2009), and it regulates the expression of some genes related to low temperature (Vornanen et al., 2005). Y-box binding protein 1 (YB-1) is a highly conserved protein that widely exists in bacteria, plants, and vertebrates (Swamynathan et al., 1998). Its conserved region can not only bind to DNA and RNA, but it also can bind to other proteins that interact with transcription factors, thereby affecting the expression and regulation of related genes. It is a very important protein in the process of body regulation (Kloks et al., 2002; Eliseeva et al., 2011). As a stress response gene, YB-1 responds to a variety of stresses in different species (Rauen et al., 2016; Li et al., 2018). Several previous studies of HMGB1 and YB-1 have focused on fish immunity (Cai et al., 2014; Xie et al., 2014; Cai X. et al., 2018; He et al., 2019).

The same gene often shows different expression in different tissues in the same organism. This has been reported in livestock (Schwerin et al., 1999; Chen et al., 2011; Ahmadi et al., 2020; Li et al., 2020), plants (Ling et al., 2018), fish (Li et al., 2008; Sun, 2017; Yu et al., 2017), and even humans (Karges et al., 1994). To study the mechanism of low temperature tolerance of *T. rubripes*, Sun (2017) used quantitative real-time PCR to analyze the expression of AFP, CIRP, HMGB1, and YB-1 in the liver, spleen, kidney, brain, heart, intestine, muscle, gonad, and skin of fish cultured under different temperature conditions (18, 13, 8, and 5°C). They reported that the expression levels of the four genes differed among tissues and temperatures. The differential expression of the same gene in different tissues could be due to the genotype effect, tissue effect, and/or genotype × tissue interactions. However, the genetic mechanisms responsible for this differential expression have not been reported to date.

In this study, an additive main effects and multiplicative interaction (AMMI) (Gollob, 1968) and genotype main effects and genotype × environment interaction (GGE) biplot analysis (Yan and Kang, 2003) was used to analyze genotype × tissue interactions affecting expression of the AFP1, CIRP, YB-1, and HMGB1 genes in *T. rubripes* cultured under different low temperature conditions. The purpose of this study was to identify the genetic mechanisms at work for the four genes in different tissues in *T. rubripes* under different low temperature conditions in order to provide a reference for formulating a breeding plan to cultivate cold-resistant varieties of *T. rubripes*.

## 2 Materials and Methods

### 2.1 Experimental Materials

The experimental *T. rubripes* came from Tianzheng Industrial Company Limited, Dalian, China. We selected 360 young fish (body length 15 ± 0.5 cm) with strong physique, no damage, and good vitality, and cultured them for 2 weeks in 12 experimental barrels (500 L, each containing 30 fish) equipped with static water aeration. The water was changed once per day.

For the experiment, four temperatures (18, 13, 8, and 5°C) were set, with triplicate barrels for each temperature. After the temporary cultivation, we used acute water exchange and cooling to bring the barrels to the correct temperature. While discharging the seawater, seawater previously adjusted to the experimental temperature was added until the temperature reached the target temperature. Yameiguang 411-H titanium heaters (GermayHeater Co., Ltd., Hefei, Anhui, China) were used to control the temperature, and water temperature readings were taken every 2 h. The fish were not fed during the experiment, and the experiment ended after 24 h of stress. Three fish were taken from each breeding barrel. After being anesthetized with 200 mg/L of MS222 (tricaine methane sulfonate) (Maya Reagent, China), each fish was dissected on ice to obtain the liver, spleen, kidney, brain, heart, intestine, muscle, skin, and gonad. Tissues were immersed in 10 times the volume of RNA preservation solution (Tiangen Biotech Co., Ltd., Beijing, China) and stored at –80°C after storage at 4°C for 24 h. Yellow Sea Fisheries Research Institute, CAFS ethics committee approved the study (Decision no: YSFRI-2021023).

### 2.2 Extraction of Total RNA and Construction of the cDNA Library

Total RNA extraction kits (Tiangen Biotech Co., Ltd., Beijing, China) were used to extract RNA. Agarose gel electrophoresis was used to evaluate RNA quality, and UV spectrophotometry was used to detect the concentration of RNA. We used 1 µg of total RNA to synthesize the first strand cDNA according to the instructions of the transcript reverse transcription kit (Tiangen Biotech Co., Ltd., Beijing, China), and we froze the product at –80°C for use in the next experiment.

### 2.3 Real Time Fluorescence Quantitative PCR (RT-qPCR)

We used RT-qPCR to detect the expression of the four target genes and the reference gene (β-actin). Quantitative primers were designed using primer express 3.0 and synthesized by Sangon Biotech Co., Ltd. (Shanghai, China). The primer sequences are shown in Table 1. The 20 µL reaction volume contained the following: 10 µL of 2 × SuperReal PreMix Plus, 0.4 µL of 50 × ROX Reference Dye, 7.4 µL of RNase-Free ddH2O, 0.6 µL of each primer (upstream primer F and downstream primer R), and 1 µL of CDNA template. The RT-qPCR reaction was carried out on the ABI StepOnePlus platform (Applied Biosystems, Shanghai, China). The PCR reaction procedure was pre-denaturation at 94 °C for 30 s followed by two-step PCR (94°C for 5 s, 60°C for 30 s, 40 cycles). After the reaction, the dissolution curve was drawn to ensure the specificity and accuracy of amplification.

**TABLE 1:** Primers of AFP, CIRP, HMGB1, YB-1, and β-actin used for RT-qPCR.

Primer Name	Primer Sequence (5′-3′)
β-actin F	ATC​GTT​GGT​CGC​CCC​AGG​CAC​C
β-actin R	CTC​CTT​GAT​GTC​AGC​ACG​ATT​TC
AFP-F	TCA​CGA​ACG​GAG​GTC​TTT​CT
AFP-R	TGC​CAC​TTG​TTT​GGC​TTG​TA
CIRP-F	ATG​GCG​ACA​GGA​GTT​ATG​GT
CIRP-R	GTT​CGT​ATC​CAC​CCT​GCA​TC
HMGB1-F	GAC​AAG​GAC​ATC​GTT​GCG​TA
HMGB1-R	ATC​CTC​GTC​GTC​ATC​GTC​TT
YB-1-F	AGA​GGC​TTC​CGA​CCA​AGA​TT
YB-1-R	GTT​GGT​TCT​GAC​CAC​CTT​CG

### 2.4 Data Processing

The expression patterns of the four genes in the nine tissues were analyzed using the 2^–ΔΔCt^ method with β-actin as the reference gene.

### 2.5 Data Analysis

#### 2.5.1 Split-Split-Plot Analysis of Variance

Refer to Piepho and Edmondson (2018), this experiment was laid out as a split-split-plot design, with temperature as the main-plot factor with the four temperature gradients (18, 13, 8, and 5°C) assigned to four main plots in each of three complete replicate blocks, tissue as the sub-plot (or split-plot) factor with the nine tissues (liver, spleen, kidney, brain, heart, intestine, muscle, gonad, and skin) assigned to nine sub-plots within each main plot and cold resistant genes as the sub-sub-plot (or split-split-plot) factor with the four cold resistant genes (AFP1, CIRP, YB-1, and HMGB1) assigned to individual sub-sub-plots within each sub-plot. The split-split-plot analysis model is written according Eq 1.

$$y_{ihjk}=\mu +b_{k}+d_{ihj}+f_{ik}+g_{ihk}+e_{ihjk}$$ (1)

where $y_{ihjk}$ is the expression of the *i*th temperature treatment for the *h*th tissue and *j*th cold resistant gene in the *k*th complete block, μ is a general intercept, $b_{k}$ is the effect of the *k*th block, $d_{ihj}$ is the *ihj*-th treatment effect, $f_{ik}$ is the main-plot error associated with the *k*th block and *i*th temperature gradient, assumed to be random with zero mean and variance $\sigma_{f}^{2}$ , $g_{ihk}$ is the sub-plot error associated with the *k*th block, *i*th temperature and *h*th tissue, assumed to be random with zero mean and variance $\sigma_{g}^{2}$ , and $e_{ihjk}$ is a residual sub-sub-plot error with zero mean and variance $\sigma^{2}$ .

#### 2.5.2 AMMI Analysis

The AMMI model for the *g*th genotype (AFP1, CIRP, YB-1, and HMGB1) in the *e*th tissue (brain, heart, intestine, kidney, liver, muscle, spleen, skin, and gonad) is written according Eq 2.

$$y_{ge}=\mu +\alpha_{g}+\beta_{e}+\sum_{i=1}^{N}\lambda_{n}\gamma_{gn}\delta_{en}+\theta_{ge}$$ (2)

where $y_{ge}$ is the expression of the four genotypes *g* in tissue *e*; μ is the grand mean; $\alpha_{g}$ is the average deviation of genotypes (the average value of each genotype minus the grand average value); $\beta_{e}$ is the average deviation of the tissue (the average of each tissue minus the grand average); $\lambda_{n}$ is the eigenvalue of the *n*th interaction principal component axis (IPCA); $\gamma_{gn}$ is the genotype principal component score of the *n*th principal component; $\delta_{en}$ is the tissue principal component score of the *n*th principal component; *N* is the total number of principal component axes; and $\theta_{ge}$ is the residual.

#### 2.5.3 GGE Biplot Analysis

GGE biplot analysis can reveal the complex interactions between different factors (Yan, 1999; Yan, 2001; Yan and Holland, 2010). The gene expression data obtained from different tissues were sorted into a two-way table including genes expression and tissue, in which each value is the average value of the expression of the corresponding gene in the corresponding tissue (i.e., the phenotype value ( $y_{ge}$ )). The GGE biplot analysis model is written according Eq 3.

$$y_{ge}=\mu +\beta_{e}+\lambda_{1}\gamma_{g1}\delta_{e1}+\lambda_{2}\gamma_{g2}\delta_{e2}+\theta_{ge}$$ (3)

where $y_{ge}$ is the trait mean expression for genotype *g* in tissue *e*; μ is the grand mean; $\beta_{e}$ is the main effect of tissue *e*; $\mu +\beta_{e}$ is the mean expression across all genotypes in tissue *e*; $\lambda_{1}$ and $\lambda_{2}$ are the singular values for the first and second PCs (PC1 and PC2), respectively; $\gamma_{g1}$ and $\gamma_{g2}$ are eigenvectors of genotype *g* for PC1 and PC2, respectively; $\delta_{e1}$ and $\delta_{e2}$ are eigenvectors of tissue *e* for PC1 and PC2, respectively; and $\theta_{ge}$ is the residual associated with genotype *g* in tissue *e*.

The split-split-plot analysis, AMMI and GGE biplot analyses were performed using the DPS Data Processing System (Tang, 2007).

## 3 Results

Table 2 shows the expression of AFP1, CIRP, YB-1, and HMGB1 in different tissues from fish cultured at different temperatures.

**TABLE 2:** Expression of AFP1, CIRP, YB-1, and HMGB1 in different tissues from fish cultured at different temperatures (means ± standard deviations).

Tissue	Gene	Expression			
		5°C	8°C	13°C	18°C
Brain	AFP1	1066.966 ± 58.627	684.934 ± 160.236	155.071 ± 10.673	33.485 ± 3.920
	CIRP	19.983 ± 8.395	7.417 ± 2.371	18.696 ± 6.166	7.302 ± 1.900
	HMGB1	86.856 ± 33.051	684.411 ± 91.780	104.362 ± 7.083	28.043 ± 2.521
	YB-1	35.377 ± 7.525	2.077 ± 0.299	29.748 ± 6.091	14.051 ± 0.647
	AFP1	6293.382 ± 351.457	1499.415 ± 286.439	433.506 ± 50.107	40.844 ± 5.496
Heart	CIRP	36.390 ± 5.614	46.828 ± 15.878	12.978 ± 2.688	12.447 ± 2.165
	HMGB1	30.119 ± 3.246	371.701 ± 94.342	19.716 ± 2.299	16.091 ± 1.397
	YB-1	181.004 ± 50.614	26.619 ± 3.228	111.872 ± 13.355	71.776 ± 11.511
	AFP1	1282.112 ± 113.425	1293.809 ± 287.443	313.135 ± 27.137	49.393 ± 1.630
Intestine	CIRP	7.494 ± 1.168	12.797 ± 0.624	8.024 ± 0.397	1.012 ± 0.193
	HMGB1	3.419 ± 0.285	70.688 ± 10.000	13.614 ± 0.943	1.002 ± 0.093
	YB-1	19.524 ± 4.315	5.130 ± 3.108	32.797 ± 4.534	4.633 ± 0.599
	AFP1	2619.441 ± 138.213	2607.893 ± 763.005	273.210 ± 43.607	138.641 ± 18.764
Kidney	CIRP	13.113 ± 2.094	7.594 ± 1.847	19.429 ± 1.665	6.787 ± 0.517
	HMGB1	7.102 ± 0.812	69.846 ± 7.990	16.645 ± 0.372	4.534 ± 0.509
	YB-1	16.438 ± 4.316	1.232 ± 1.149	50.748 ± 8.688	9.875 ± 0.626
	AFP1	1794.711 ± 268.371	489.156 ± 39.355	365.993 ± 88.821	57.209 ± 29.476
Liver	CIRP	13.519 ± 7.766	5.962 ± 1.397	21.564 ± 6.086	9.331 ± 2.064
	HMGB1	18.601 ± 1.117	89.556 ± 13.502	96.255 ± 10.374	17.639 ± 0.471
	YB-1	28.098 ± 1.714	5.407 ± 1.882	68.932 ± 7.887	53.148 ± 8.477
	AFP1	6116.457 ± 1378.037	1811.152 ± 690.646	387.966 ± 27.999	194.620 ± 36.069
Muscle	CIRP	89.788 ± 57.712	40.804 ± 11.521	16.792 ± 1.359	15.674 ± 0.434
	HMGB1	66.614 ± 5.001	121.446 ± 5.530	50.482 ± 1.620	35.631 ± 3.744
	YB-1	3514.809 ± 638.609	509.614 ± 165.296	2000.654 ± 86.534	2710.596 ± 483.487
	AFP1	3512.656 ± 31.531	3179.010 ± 33.714	388.093 ± 49.514	91.363 ± 9.527
Spleen	CIRP	19.201 ± 3.057	12.285 ± 4.973	5.310 ± 0.349	3.982 ± 0.129
	HMGB1	15.677 ± 1.745	101.118 ± 21.340	9.094 ± 0.752	4.701 ± 0.398
	YB-1	29.009 ± 2.606	0.225 ± 0.148	20.453 ± 2.186	5.817 ± 1.006
	AFP1	7400.707 ± 868.984	893.144 ± 54.822	441.387 ± 55.797	145.049 ± 11.767
Skin	CIRP	15.086 ± 0.933	18.135 ± 0.696	16.181 ± 4.398	4.378 ± 0.133
	HMGB1	11.748 ± 0.850	59.467 ± 5.569	29.777 ± 3.075	13.118 ± 2.621
	YB-1	151.941 ± 40.267	66.343 ± 14.264	66.961 ± 8.481	75.281 ± 5.565
	AFP1	2933.386 ± 245.377	449.5484 ± 128.829	4.213 ± 0.471	1.025 ± 0.286
Gonad	CIRP	9.144 ± 0.729	7.027 ± 1.484	2.295 ± 0.246	2.733 ± 0.260
	HMGB1	22.158 ± 3.001	35.308 ± 4.753	3.276 ± 0.243	3.623 ± 0.611
	YB-1	21.180 ± 4.578	1.818 ± 0.545	2.847 ± 0.326	1.004 ± 0.116

### 3.1 Split-Split-Plot Analysis of Variance

The results of the split-split-plot analysis of variance were listed in Table 3. Table 3 showed that the *p* values of factors temperature, tissue, gene, temperature × tissue, temperature × gene, tissue × gene, and temperature × tissue ×gene were 0.0143, 0, 1E-07, 0, 1E-07, 1.887E-06 and 0.0152,694, respectively, indicating that the expression of the four cold resistant genes was significantly (*p* < 0.05) affected by temperature and temperature × tissue ×gene interaction, and was highly significantly (*p* < 0.01) affected by tissue, gene, temperature × tissue, temperature × gene and tissue × gene interaction (Table 3).

**TABLE 3:** Split-split-plot analysis of variance for *T. rubripes* cold resistant experiment with four cold resistant genes, four temperature gradients and nine tissues.

Source of Variation	Sum of Square	Degrees of Freedom	Mean Square	*F*-Value	*p*-value
Blocks (replicates)	40252485	5	8050497		
Temperature	29812447	3	9937482.5	4.9102333*	0.0143
Main-plot error	30357466	15	2023831		
Tissue	17330089	8	2166261.1	9.1797**	0
Temperature × tissue	17897111	24	745712.94	3.16**	0
Split-plot error	37757296	160	235983.1		
Gene	65742384	3	21914128	52.140,554**	1E-07
Temperature × gene	80367779	9	8929753.2	21.246,671**	1E-07
Tissue × gene	30951837	24	1289659.9	3.0685,036**	1.887E-06
Temperature × tissue ×gene	43351378	72	602102.48	1.4325898*	0.0152,694
Split-split-plot error	226956339	540	420289.52		

Notes: Asterisks denote that correlations were significant at *p < 0.05 and **p < 0.01.

**FIGURE 1:**
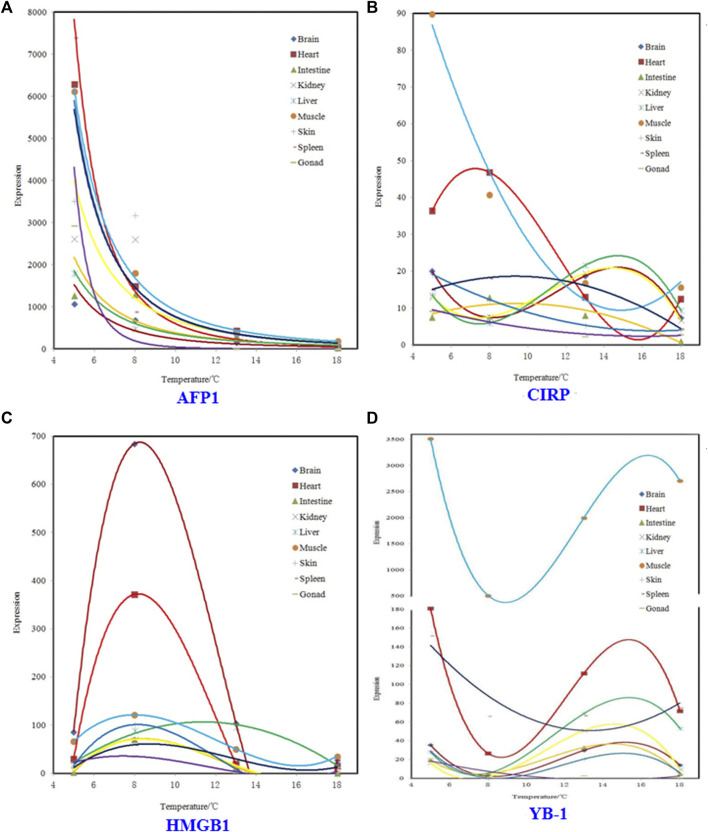
Regression models for relation between expression and temperature for four genes.

Regression models for relation between expression and temperature for four genes were showed in Figure 1. In Figure 1, panel A, all regression models are power functions. In Figure 1, panel B, regression models for brain, intestine, kidney, and liver are three-degree polynomial, and that for muscle, skin, spleen, and gonad are second-degree polynomial. In Figure 1, panel C, except that liver is second-degree polynomial, other tissues are three-degree polynomial. In Figure 1, panel D, except that spleen and gonad are second-degree polynomial, other tissues are three-degree polynomial.

### 3.2 AMMI Analysis

The results of AMMI analysis showed that the expression of the four genes was significantly affected by genotype, tissue, and genotype × tissue interactions at different temperatures (Table 4).

**TABLE 4:** Results of AMMI analysis of expression of the four genes in different tissues from fish cultured at different temperatures.

Source of	5°C					
Variation	*df*	SS	MS	*F*	Prob	% Of Total SS
Total	107	423623818.3	3959101.1			
Treatment	35	416897702.1	11911363	127.51**	0	
Gene	3	251594453.7	83864818	897.73**	0	59.3910
Tissue	8	56355829.37	7,044,478.7	75.408**	0	13.3032
Interaction	24	108,947,419.1	4,539,475.8	48.593**	0	25.7179
IPCA1	10	90,456,102.53	9,045,610.3	96.829**	0	83.0273
IPCA2	8	18,486,274.89	2,310,784.4	24.736**	0	16.9680
Residual	6	5041.6457	840.2743			
Error	72	6,726,116.19	93418.28			
**Source of**			**8 °C**			
**variation**	***df***	**SS**	**MS**	***F***	**Prob**	**% of total SS**
Total	107	63,381,780.3	592353.087			
Treatment	35	60,745,799	1,735,594.26	47.406**	0	
Gene	3	37,053,112.4	12351037.5	337.36**	0	58.460
Tissue	8	5,634,930.89	704366.361	19.239**	0	8.890
Interaction	24	18,057,755.7	752406.488	20.551**	0	28.490
IPCA1	10	16914065.4	1,691,406.54	46.199**	0	93.666
IPCA2	8	938525.287	117315.661	3.204**	0.003	5.197
Residual	6	205165.014	34194.169			
Error	72	2,635,981.37	36610.852			
**Source of**			**13 °C**			
**variation**	***df***	**SS**	**MS**	***F***	**Prob**	**% of total SS**
Total	107	12,642,216.3	118152			
Treatment	35	12,586,769.2	359622	466.982**	0	
Gene	3	1,859,582.52	619861	804.911**	0	14.709
Tissue	8	3,013,081.06	376635	489.074**	0	23.833
Interaction	24	7,714,105.65	321421	417.377**	0	61.018
IPCA1	10	7,373,491.72	737349	957.474**	0	95.584
IPCA2	8	328220.754	41027.6	53.275**	0	4.254
Residual	6	12393.176	2065.53			
Error	72	55447.070	770.098			
**Source of**			**18 °C**			
**variation**	***df***	**SS**	**MS**	***F***	**Prob**	**% of total SS**
Total	107	21594750.1	201820			
Treatment	35	21,121,069.5	603459	91.726**	0	
Gene	3	1,829,837.74	609946	92.712**	0	8.473
Tissue	8	5,404,080.57	675510	102.678**	0	25.024
Interaction	24	13,887,151.2	578631	87.952**	0	64.308
IPCA1	10	13,847,644.7	1,384,764	210.486**	0	99.715
IPCA2	8	38976.135	4872.02	0.740	0.655	0.280
Residual	6	530.417	88.4028			
Error	72	473680.549	6578.9			

Notes: one *df*: degrees of freedom, SS: sum of squares, MS: mean squares, F: test statistic, Prob: probability.

2 **: significant at 1% probability level.

At 5°C, the AMMI analysis of variance indicated that 59.3910%, 13.3032%, and 25.7179% of the total sum of squares (SS) were attributable to genotype, tissue effects, and genotype × tissue interactions, respectively. IPCA1 and IPCA2 were obtained, which contributed to 83.0273 and 16.9680% of the genotype × tissue interactions, respectively.

At 8°C, the AMMI analysis of variance indicated that 58.460%, 8.890%, and 28.490% of the total SS were attributable to genotype, tissue effects, and genotype × tissue interactions, respectively. IPCA1 and IPCA2 contributed 93.666 and 5.197% of the genotype × tissue interactions, respectively.

At 13 °C, the AMMI analysis of variance indicated that 14.709%, 23.833%, and 61.018% of the total SS were attributable to genotype, tissue effects, and genotype × tissue interactions, respectively. IPCA1 and IPCA2 contributed 95.584 and 4.254% of the genotype × tissue interactions, respectively.

At 18 °C, the AMMI analysis of variance indicated that 8.473%, 25.024%, and 64.308% of the total SS were attributable to genotype, tissue effects, and genotype × tissue interactions, respectively. IPCA1 and IPCA2 contributed 99.715 and 0.280% of the genotype × tissue interactions, respectively.

### 3.3 GGE Biplot Analysis

GGE biplot analysis was carried out based on the mean expression of the four genes in the nine tissues at different culture temperatures. The “relationship among different tissues,” “which-won-where,” and “high expression and expression stability” view of the GGE biplots and the “concentric circles” view of the GGE biplots were drawn based on the conclusions of the GGE biplot analysis.

**FIGURE 2:**
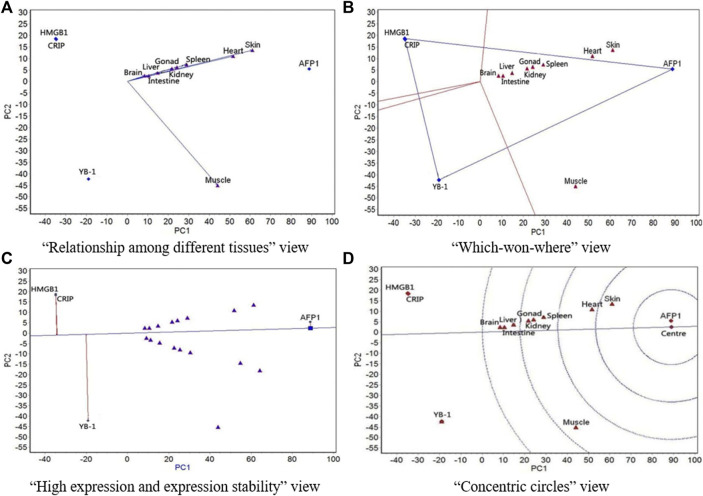
The GGE biplot for expression of four resistance genes to low temperature (AFP1, CIRP, YB-1, and HMGB1) in different tissues at 5°C. **(A)** “Relationship among different tissues” view. **(B)** “Which-won-where” view. **(C)** “High expression and expression stability” view. **(D)** “Concentric circles” view.

**FIGURE 3:**
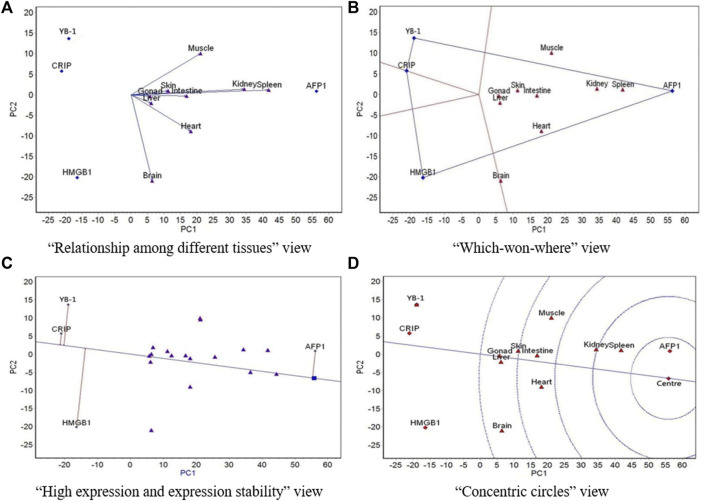
The GGE biplot for expression of four resistance genes to low temperature (AFP1, CIRP, YB-1, and HMGB1) in different tissues at 8°C. **(A)** “Relationship among different tissues” view. **(B)** “Which-won-where” view. **(C)** “High expression and expression stability” view. **(D)** “Concentric circles” view.

**FIGURE 4:**
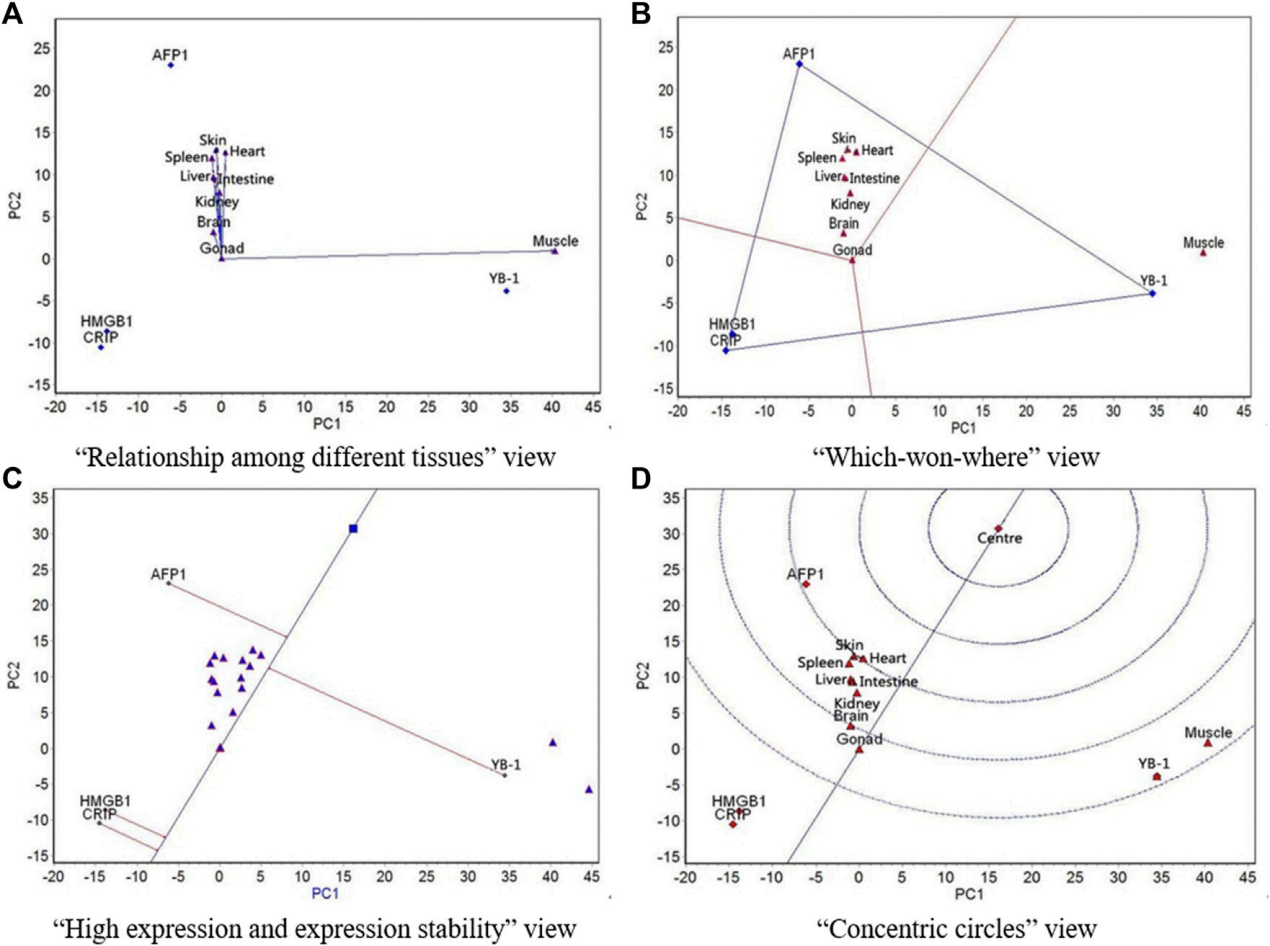
The GGE biplot for expression of four resistance genes to low temperature (AFP1, CIRP, YB-1, and HMGB1) in different tissues at 13°C. **(A)** “Relationship among different tissues” view. **(B)** “Which-won-where” view. **(C)** “High expression and expression stability” view. **(D)** “Concentric circles” view.

**FIGURE 5:**
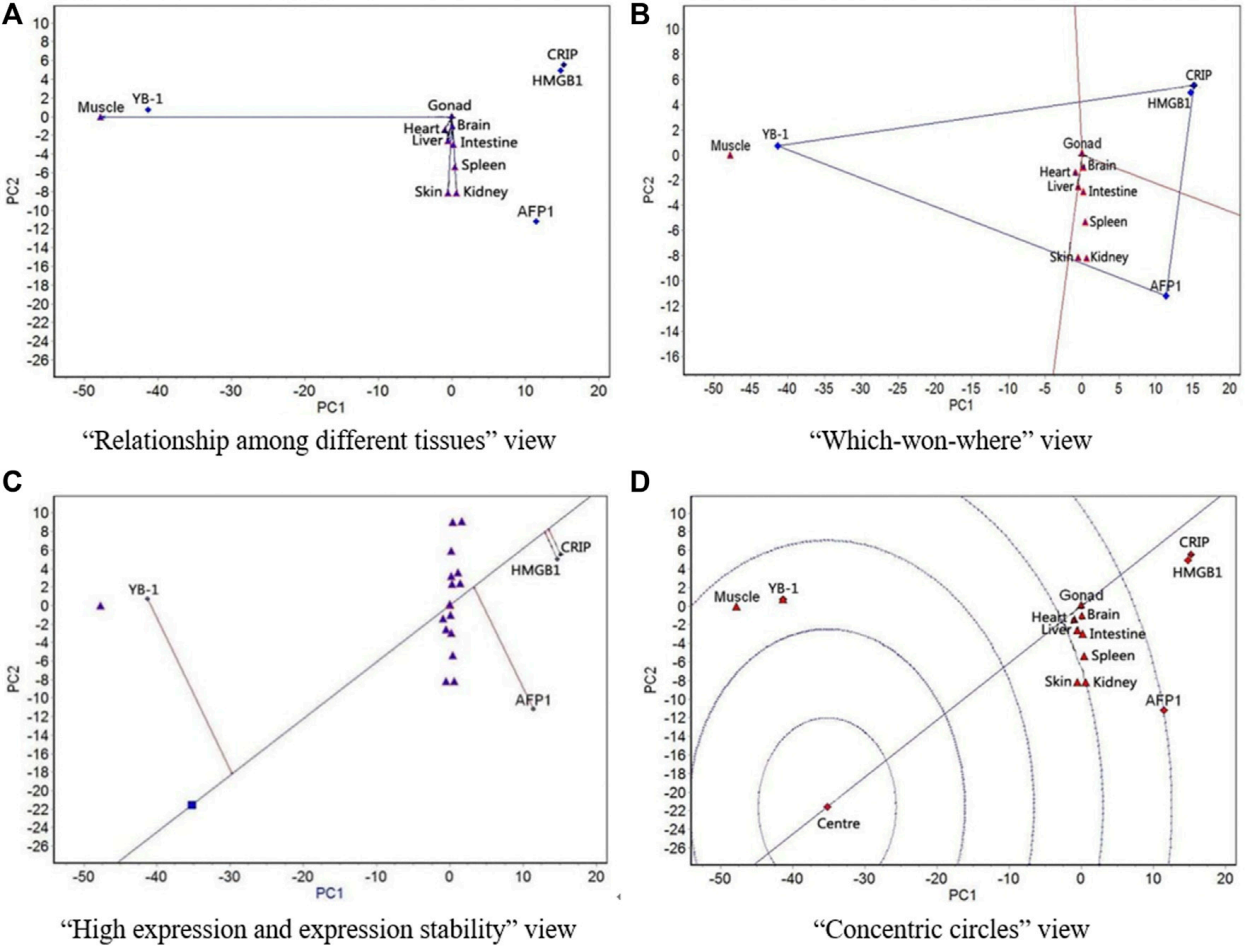
The GGE biplot for expression of four resistance genes to low temperature (AFP1, CIRP, YB-1, and HMGB1) in different tissues at 18°C. **(A)** “Relationship among different tissues” view. **(B)** “Which-won-where” view. **(C)** “High expression and expression stability” view. **(D)** “Concentric circles” view.

The GGE biplots of the “relationship among different tissues” (Figures 2–5, panel A) mainly focuses on the similarity of genes expression among tissues. The cosine of the angle between two line segments is the correlation coefficient of the gene expression in two tissues. An angle <90° indicates a positive correlation and that the expression of genes in the two tissues is similar, whereas an angle >90° indicates a negative correlation and that the expression of genes in the two tissues is in the opposite ranking. A 90° angle indicates that the gene expression in two tissues is not related. The length of the line segment indicates the ability of the tissue to distinguish gene expression: the longer the line segment, the stronger the ability to distinguish gene expression (Tang, 2007).

The “which-won-where” view of GGE biplots (Figures 2–5, panel B) divides the experimental regions according to the interaction between gene and tissue, and it reveals genes with the highest expression level in each region. The gene located at the top corner of the polygon in each region is the gene with the highest expression in this region (Tang, 2007).

The “high expression and expression stability” view of the GGE biplots (Figures 2–5, panel C) identifies genes with high and stable expression. The direction of the transverse oblique line to “ideal gene” is the approximate average expression of genes in all tissues: the closer to the ideal variety, the higher the average gene expression. The straight line perpendicular to the transverse slash represents the tendency of the gene × tissue interaction: the greater the deviation of the vertical line from the transverse oblique line, the more unstable the gene expression (Tang, 2007).

Finally, the GGE biplots with concentric circles (Figures 2–5, panel D) comprehensively evaluate the high expression and expression stability based on the distance of various genes to the central point of the genes: the smaller the distance, the higher and more stable the expression of the gene (Tang, 2007).

At 5°C, the relationship among different tissues (Figure 2A) showed that the angles between all tissues except for muscle were very small (the correlations among the eight tissues were highly positive), which indicates that expression rankings of all four genes was basically the same in the eight tissues. Muscle had the longest line segment length, which indicates that it had the strongest ability to distinguish expression of the four genes, followed by skin. The “which-won-where” view (Figure 2B) showed that all nine tissues belonged to a region, where AFP1 had the highest expression. The “high expression and expression stability” view of the GGE biplot (Figure 2C) showed that the expression of AFP1 was highest, followed by YB-1, CIRP, and HMGB1. AFP1 had the most stable expression, followed by CIRP, HMGB1, and YB-1. The concentric circles biplot (Figure 2D) showed that AFP1 had the best expression and stability, followed by YB-1, CIRP, and HMGB1.

At 8 °C, the angles between all tissues except for heart, brain, and muscle were very small (the correlations among the six tissues were highly positive), which indicates that expression rankings of all four genes was basically the same in the six tissues (Figure 3A). There was a low positive correlation of heart and muscle with these six tissues, and there was a negative correlation between brain and muscle. The spleen had the strongest ability to distinguish expression of the four genes, followed by the kidney. The nine tissues belonged to a region, and AFP1 had the highest expression in this region (Figure 3B). The expression of AFP1 was highest, followed by HMGB1, YB-1, and CIRP, and the most stable expression was CIRP, followed by AFP1, YB-1, and HMGB1 (Figure 3C). AFP1 had the best expression and stability, followed by HMGB1, CIRP, and YB-1 (Figure 3D).

At 13°C, with the exception of muscle, the other eight tissues showed similar expression of the four genes (Figure 4A). Muscle had the strongest ability to distinguish expression rankings of the four genes, followed by skin. The nine tissues were divided into two regions, with muscle in one region and the other eight tissues in the other region; YB-1 had the highest expression in the muscle region, whereas AFP1 had the highest expression in the other region (Figure 4B). The expression of AFP1 was highest, followed by YB-1, HMGB1, and CIRP, and the most stable expression was CIRP, followed by HMGB1, AFP1, and YB-1 (Figure 4C). AFP1 had the best expression and stability, followed by YB-1, HMGB1, and CIRP (Figure 4D).

At 18 °C, all tissues except for muscle showed similar expression of the four genes (Figure 5A). Muscle had the strongest ability to distinguish expression rankings of the four genes, followed by skin. The nine tissues were divided into two regions, with gonad, muscle, and heart in one region and the other six tissues in the other region; YB-1 had the highest expression in the gonad-muscle-heart region, and AFP1 had the highest expression in the other region (Figure 5B). The expression of YB-1 was the highest, followed by AFP1, HMGB1, and CIRP, and the most stable expression was CIRP, followed by HMGB1, AFP1, and YB-1 (Figure 5C). YB-1 had the best expression and stability, followed by AFP1, HMGB1, and CIRP (Figure 5D).

## 4 Discussion

### 4.1 Split-Split-Plot Analysis of Variance

The results of split-split-plot analysis of variance showed that water temperature has a significant (*p* < 0.05) effect on the expression of *T. rubripes* cold resistant genes, while tissue × gene interaction has a highly significant (*p* < 0.01) effect on it, which indicates that it is of great significance to carry out dissection of genotype × tissue interactions for *T. rubripes* cold resistant genes under different low-temperature conditions for determining temperature range of low temperature tolerance experiments, selecting tissues for gene expression analysis, and screening for antifreeze genes with high expression and high stability in cold-resistant strains of *T. rubripes.*

On the whole, the expression of AFP1, in the nine tissues, were power functions with temperature, that of CIRP were two- and three-degree polynomial with temperature, and that of HMGB1 and YB-1 were three-degree polynomial with temperature, which showed that different cold tolerant genes showed unique expression patterns under the same temperature gradient. Among them, the expression mechanism of AFP1 is quite different from that of the other three genes, while the expression mechanisms of HMGB1 and YB-1 are relatively consistent.

### 4.2 AMMI Analysis

The results of AMMI analysis showed that the expression of the four genes was significantly affected by genotype, tissue, and genotype × tissue interactions at different culture temperatures. However, at different temperatures, the contributions of gene, tissue, and interactions to the total variation in gene expression differed, but followed certain patterns. As temperature decreased, the gene effect increased gradually and the genotype × tissue interaction decreased gradually. This may be because it is easier to express cold tolerance genes at lower the temperature. In addition, the gene effect at 18 and 13 °C was significantly lower than that at 8 and 5°C, whereas the interaction at 18 and 13 °C was significantly higher than that at 8 and 5°C. The higher two temperatures may be in the range at which antifreeze gene expression is inactive, while it might be active at 8 and 5 °C. At 18, 13, and 8 °C, the tissue effect decreased gradually with decreasing temperature, but it increased at 5 °C. However, the tissue effect at 18 and 13 °C was significantly higher than that at 8 and 5 °C. We speculate that 8 °C may be in the temperature range in which inactive expression becomes active, and therefore this temperature affects the distribution of the three effects. It also may also be the key regulation point for *T. rubripes* to deal with low temperature stress. Temperature that is too low will cause regulation disorder, thus 8 °C may be a relevant basis for studying the regulation mechanism of the low temperature response of *T. rubripes*.

### 4.3 GGE Biplot Analysis

The GGE biplot analysis of the “relationship among different tissues” (Figures 2–5, panel A) showed that at 5, 13, and 18 °C, the expression of AFP1, CIRP, YB-1, and HMGB1 was highly positively correlated in all tissues except muscle, which indicates that the expression of the four genes in the liver, spleen, kidney, brain, heart, intestine, gonad, and skin was basically the same at these three temperatures. At 8 °C, the expression of the four genes in the brain, heart, and muscle was lower than that in the liver, spleen, kidney, intestine, gonad, and skin. At all four temperatures, the ability of the nine tissues to distinguish the expression of the four genes showed a law similar to the above changes. At 5, 13, and 18 °C, muscle had the strongest ability to distinguish expression of the four genes, followed by skin, which likely is related to the important roles of skin and muscle in coping with temperature changes, as these organs are in close contact with the external environment. At 8 °C, the spleen had the strongest ability to distinguish expression of the four genes, followed by the kidney. One possible explanation for the observed difference at this temperature is that 8 °C may be in the transition zone at which inactive gene expression becomes active.

The “which-won-where” view of the GGE biplot (Figures 2–5, panel B) showed that at 5 and 8 °C, all nine tissues belonged to the same region, in which AFP1 had the highest expression. At 13 °C, the nine tissues were divided into two regions, with muscle in one region and the other eight tissues in the other one; YB-1 had the highest expression in the muscle region, and AFP1 had the highest expression in the other region. At 18 °C, the nine tissues were divided into two regions, with the gonad, muscle, and heart in one region and the other six tissues in the other region; YB-1 had the highest expression in the gonad-muscle-heart region, and AFP1 had the highest expression in the other region. In the gonad-muscle-heart region, the gonad and heart were located very close to the other region and far away from the muscle, which shows that the regions at 18 and 13 °C were basically the same. Thus, on the whole, the region for 5 and 8 °C was similar and that for 13 and 18 °C was similar. This may be related to the different levels of antifreeze gene expression activity at 18 °C/13 and 8 °C/5 °C. Further analysis showed that at 5, 13, and 18 °C, muscle was far removed from the other eight tissues, while the other eight tissues were situated close to each other. At 8 °C, however, the pattern did not follow this structure, which shows that the regional division of other three temperatures was relatively consistent. Again, 8 °C is likely within the transition temperature range in which gene expression change from inactive to active.

In the “high expression and expression stability” view of the GGE biplot, the expression rankings of the four genes were similar at 5, 13, and 18 °C, but slightly different from those at 8 °C. Consistent with the other biplot views, this is likely because 8 °C is located in the transition temperature range in which gene expression changes from inactive to active. At 13 and 18 °C, the expression stability of the four genes was the same, and their stability at 5 and 8 °C was even more similar than at 13 and 18 °C. This is likely due to the different levels of antifreeze gene expression activity at 18 °C/13 vs 8 °C/5 °C. The expression of AFP1 was second highest at 18 °C and highest at the other three temperatures, and AFP1 expression became more and more stable as temperature decreased. The expression of CIRP ranked third at 5 °C and fourth at the other three temperatures, whereas the expression stability ranked second at 5 °C and first at the other three temperatures.

The “concentric circles” view of the GGE biplot illustrates the comprehensive evaluation of the expression amount and stability of the four genes at different temperatures. Both expression amount and stability were more similar at 5, 13, and 18 °C. AFP1 showed the best expression and stability among the four genes, as it ranked second at 18 °C and first at the other three temperatures.

## 5 Conclusion

The results of split-split-plot analysis of variance showed that water temperature has a significant effect on the expression of *T. rubripes* cold resistant genes, while tissue × gene interaction has a highly significant effect on it. On the whole, the expression of AFP1, in the nine tissues, were power functions with temperature, that of CIRP were two- and three-degree polynomial with temperature, and that of HMGB1 and YB-1 were three-degree polynomial with temperature.

The results of AMMI analysis showed that expression of AFP1, CIRP, YB-1, and HMGB1 was significantly affected by genotype, tissue, and genotype × tissue interactions at different temperatures and that the contributions of these effects followed certain trends. The patterns suggest that: 1) as temperature decreased, the gene effect increased gradually and the genotype × tissue interaction decreased gradually; 2) 18 and 13 °C are in the temperature range in which antifreeze gene expression is inactive, whereas expression is active at 8 and 5 °C; and 3) 8 °C may be in the transition temperature range in which gene expression changes from inactive to active.

The results of the GGE biplot analysis showed that at all temperatures except 8 °C, the expression of AFP1, CIRP, YB-1, and HMGB1 was highly positively correlated in all tissues except muscle, thus the expression rankings in those eight tissues were basically the same at the three temperatures. At 5, 13, and 18 °C, muscle had the strongest ability to distinguish expression of the four genes, followed by skin, which likely is because these two organs are in close contact with the changing external environment. At 5 and 8 °C, the nine tissues were clustered into one region and at 13 and 18 °C they were divided into two regions, possibly due to different levels of antifreeze gene expression activity at 18 °C/13 vs 8 °C/5 °C. At 5, 13, and 18 °C, the expression rankings of the four genes were similar, and the expression stability of the genes was the same at 18 °C/13 °C and at 8 °C/5 °C, possibly due to different levels of antifreeze gene expression at higher vs lower temperatures. Among the four genes, AFP1 showed the best expression and stability.

These findings can be used to develop cold-resistant strains of *T. rubripes*. When carrying out low temperature tolerance breeding, it is crucial to identify the temperature range for low temperature tolerance experiments. We identified 8 °C/5 °C as the suitable temperature for such experiments for *T. rubripes*. Due to the interaction between genes and the tissues in which they are expressed, the expression of the same gene differs among tissues. Therefore, in molecular breeding, selecting the best tissue for studying the expression of a specific gene is important. We found that muscle and skin were the preferred tissues for studying antifreeze gene expression in *T. rubripes*. Screening for antifreeze genes with high expression and high stability is the key to the success of low temperature tolerance breeding. Among the four antifreeze genes evaluated in this study, AFP1 had the best expression and stability.

## Data Availability

The original contributions presented in the study are included in the Supplementary Material, further inquiries can be directed to the corresponding author.

## References

[B1] Abdel-Tawwab M. Wafeek M. (2014). Influence of Water Temperature and Waterborne Cadmium Toxicity on Growth Performance and Metallothionein-Cadmium Distribution in Different Organs of Nile tilapia, *Oreochromis niloticus* (L.). J. Therm. Biol. 45, 157–162. 10.1016/j.jtherbio.2014.09.002 25436965

[B2] Ahmadi F. Abadi A.-R. Bazi Z. Movafagh A. (2020). Novel Study of Model-Based Clustering Time Series Gene Expression in Different Tissues: Applications to Aging Process. Curr. Aging Sci. 13, 178–187. 10.2174/1874609812666191015140449 31749443

[B3] Al-Fageeh M. B. Smales C. M. (2006). Control and Regulation of the Cellular Responses to Cold Shock: the Responses in Yeast and Mammalian Systems. Biochem. J. 397 (2), 247–259. 10.1042/BJ20060166 16792527 PMC1513281

[B4] Cai J. Xia H. Huang Y. Lu Y. Wu Z. Jian J. (2014). Molecular Cloning and Characterization of High Mobility Group Box1 (Ls-HMGB1) from Humphead Snapper, *Lutjanus Sanguineus* . Fish Shellfish Immunol. 40 (2), 539–544. 10.1016/j.fsi.2014.08.004 25120217

[B5] Cai L. Y. Nian L. Y. Cao A. L. Wang Y. B. Guan R. F. Zhao Y. H. (2018). Research Progress in the Structure-Function Relationship of Aquatic Antifreeze Proteins and its Application in Food Processing and Storage. Sci. Technol. Food Industry 39 (22), 346–352. 10.13386/j.issn1002-0306.2018.22.060

[B6] Cai X. Gao C. Su B. Tan F. Yang N. Wang G. (2018). Expression Profiling and Microbial Ligand Binding Analysis of High-Mobility Group Box-1 (HMGB1) in Turbot (*Scophthalmus maximus* L.). Fish Shellfish Immunol. 78, 100–108. 10.1016/j.fsi.2018.04.025 29679761

[B7] Chen Y. Yang S. L. Deng W. D. Mao H. M. (2011). Differential Gene Expression in Different Tissues of Black-Bone Sheep and Normal Sheep. Asian J. Animal Veterinary Adv. 6 (4), 379–384. 10.3923/ajava.2011.379.384

[B8] Cui J. Li J. L. Wang Z. Y. Dai C. H. Liu J. L. Liu T. J. (2019). Cloning of Gene of Carrot Antifreeze Protein and its Transformation to Sugar Beet. China Beet Sugar 3, 1–4. 10.3969/j.issn.1002-0551.2019.03.001

[B9] Duman J. G. Olsen T. M. (1993). Thermal Hysteresis Protein Activity in Bacteria, Fungi, and Phylogenetically Diverse Plants. Cryobiology 30 (3), 322–328. 10.1006/cryo.1993.1031

[B10] Eliseeva I. A. Kim E. R. Guryanov S. G. Ovchinnikov L. P. Lyabin D. N. (2011). Y-box-binding Protein 1 (YB-1) and its Functions. Biochem. Mosc. 76 (13), 1402–1433. 10.1134/S0006297911130049 22339596

[B11] Gollob H. F. (1968). A Statistical Model Which Combines Features of Factor Analytic and Analysis of Variance Techniques. Psychometrika 33, 73–115. 10.1007/BF02289676 5239571

[B12] He J. Xie T.-L. Li X. Yu Y. Zhan Z.-P. Weng S.-P. (2019). Molecular Cloning of Y-Box Binding Protein-1 from Mandarin Fish and its Roles in Stress-Response and Antiviral Immunity. Fish Shellfish Immunol. 93, 406–415. 10.1016/j.fsi.2019.07.069 31369857

[B13] Hew C. Poon R. Xiong F. Gauthier S. Shears M. King M. (1999). Liver-specific and Seasonal Expression of Transgenic Atlantic Salmon Harboring the Winter Flounder Antifreeze Protein Gene. Transgenic Res. 8, 405–414. 10.1023/a:1008900812864 10767985

[B14] Hu J. W. You F. Wang Q. Wang L. J. Weng S. D. Xin M. J. (2015). Cloning and Expression Analysis of Cold-Tolerance Related Genes, CIRP and HMGB1, in *Paralichthys olivaceus* . Mar. Sci. 39 (1), 29–38. 10.11759/hykx20140304001

[B15] Islam M. A. Uddin M. H. Uddin M. J. Shahjahan M. (2019). Temperature Changes Influenced the Growth Performance and Physiological Functions of Thai Pangas *Pangasianodon Hypophthalmus* . Aquac. Rep. 13, 100179. 10.1016/j.aqrep.2019.100179

[B16] Islam M. J. Slater M. J. Bögner M. Zeytin S. Kunzmann A. (2020). Extreme Ambient Temperature Effects in European Seabass, *Dicentrarchus labrax*: Growth Performance and Hemato-Biochemical Parameters. Aquaculture 522, 735093. 10.1016/j.aquaculture.2020.735093

[B17] Karges W. J. Gaedigk R. Dosch H. M. (1994). Quantitative Analysis of Gene Expression in Different Tissues by Template-Calibrated RT-PCR and Laser-Induced Fluorescence. Genome Res. 4, 154–159. 10.1101/gr.4.3.154 7580899

[B18] Kloks C. P. A. M. Spronk C. A. E. M. Lasonder E. Hoffmann A. Vuister G. W. Grzesiek S. (2002). The Solution Structure and DNA-Binding Properties of the Cold-Shock Domain of the Human Y-Box Protein YB-1. J. Mol. Biol. 316 (2), 317–326. 10.1006/jmbi.2001.5334 11851341

[B19] Lange S. S. Vasquez K. M. (2009). HMGB1: The Jack-Of-All-Trades Protein Is a Master DNA Repair Mechanic. Mol. Carcinog. 48 (7), 571–580. 10.1002/mc.20544 19360789 PMC2856944

[B20] Li G. Wang L. Wang Y. Li H. Liu Z. Wang H. (2018). Developmental Characterization and Environmental Stress Responses of Y-Box Binding Protein 1 Gene (AccYB-1) from *Apis cerana* Cerana. Gene 674, 37–48. 10.1016/j.gene.2018.06.071 29940273

[B21] Li J. Mao S. M. Zhou D. Feng W. W. Li P. Chen D. F. (2020). The Study on mRNA Expression of MC4R and SIMI Genes in Different Tissues for Congjiang Pigs. Swine Prod. 3, 65–68. 10.3969/j.issn.1002-1957.2020.03.030

[B22] Li Y. Ding L. Li Y. Hou N. Chang Y. M. Liang L. Q. (2008). Influence on the Expression Quantity of Five Genes in the Different Tissues of Common Carps *Cyprinus carpio* under the Low Temperature. Acta Zool. Sin. 54 (3), 460–466. 10.3969/j.issn.1007-1954.2010.04.003

[B23] Ling A. Li X. Hu X. Ma Z. Wu K. Zhang H. (2018). Dynamic Changes in Polyphenol Compounds, Antioxidant Activity, and PAL Gene Expression in Different Tissues of Buckwheat during Germination. J. Sci. Food Agric. 98 (15), 5723–5730. 10.1002/jsfa.9119 29736979

[B24] Miao L. Li M. Y. Chen Y. Y. Mou H. U. Chen J. (2017). Cloning of Cold Inducible RNA-Binding Protein(CIRP) Gene in Larimichthys Crocea and its Expression Analysis under Cold Treatments. J. Fish. China 41 (4), 481–489. 10.11964/jfc.20160410364

[B25] Nishiyama H. Itoh K. Kaneko Y. Kishishita M. Yoshida O. Fujita J. (1997). A Glycine-Rich RNA-Binding Protein Mediating Cold-Inducible Suppression of Mammalian Cell Growth. J. Cell Biol. 137 (4), 899–908. 10.1083/jcb.137.4.899 9151692 PMC2139845

[B26] Piepho H. P. Edmondson R. N. (2018). A Tutorial on the Statistical Analysis of Factorial Experiments with Qualitative and Quantitative Treatment Factor Levels. J. Agro. Crop. Sci. 204 (5), 429–455. 10.1111/jac.12267

[B27] Rauen T. Frye B. C. Wang J. Raffetseder U. Alidousty C. En-Nia A. (2016). Cold Shock Protein YB-1 Is Involved in Hypoxia-dependent Gene Transcription. Biochem. Biophysical Res. Commun. 478 (2), 982–987. 10.1016/j.bbrc.2016.08.064 27524241

[B28] Raymond J. A. DeVries A. L. (1977). Adsorption Inhibition as a Mechanism of Freezing Resistance in Polar Fishes. Proc. Natl. Acad. Sci. U.S.A. 74 (6), 2589–2593. 10.1073/pnas.74.6.2589 267952 PMC432219

[B29] Schwerin M. Voigt J. Wegner J. Kuhn C. Ender K. Hagemeister H. (1999). Gene Expression in Different Tissues of Lactating Cows of Differing Metabolic Type: 1. Comparison of mRNA Patterns by the Differential Display Method. J. Anim. Physiol. Anim. Nutr. 81, 113–123. 10.1046/j.1439-0396.1999.813177.x

[B30] Sun J. H. (2017). Differential Gene Expression Analysis of Low Temperture Transcriptome and Genealogical Identification from *Takifugu rubripes* . Shanghai, China: Shanghai Ocean University. Master degree theses.

[B31] Swamynathan S. K. Nambiar A. Guntaka R. V. (1998). Role of Single‐stranded DNA Regions and Y‐box Proteins in Transcriptional Regulation of Viral and Cellular Genes. FASEB J. 12 (7), 515–522. 10.1096/fasebj.12.7.515 9576478

[B32] Tang Q. Y. (2007). DPS Data Processing System. Beijing: Science Press.

[B33] Vornanen M. Hassinen M. Koskinen H. Krasnov A. (2005). Steady-state Effects of Temperature Acclimation on the Transcriptome of the Rainbow Trout Heart. Am. J. Physiol. Regul. Integr. Comp. Physiol. 289 (4), R1177–R1184. 10.1152/ajpregu.00157.2005 15932967

[B34] Wang S. Y. Li X. K. Zhou Y. F. Wu J. H. (2012). Research Progress in Antifreeze Machanism and Genetic Engineering of Antifreeze Protein. J. Beijing Technol. Bus. Univ. Nat. Sci. Ed. 30 (2), 58–63. 10.3969/j.issn.1671-1513.2012.02.012

[B35] Wen B. Jin S.-R. Chen Z.-Z. Gao J.-Z. Wang L. Liu Y. (2017). Plasticity of Energy Reserves and Metabolic Performance of Discus Fish (*Symphysodon aequifasciatus*) Exposed to Low-Temperature Stress. Aquaculture 481, 169–176. 10.1016/j.aquaculture.2017.09.002

[B36] Xie J. Hodgkinson J. W. Li C. Kovacevic N. Belosevic M. (2014). Identification and Functional Characterization of the Goldfish (*Carassius auratus* L.) High Mobility Group Box 1 (HMGB1) Chromatin-Binding Protein. Dev. Comp. Immunol. 44 (1), 245–253. 10.1016/j.dci.2013.12.015 24406304

[B37] Xu D. You Q. Chi C. Luo S. Song H. Lou B. (2018). Transcriptional Response to Low Temperature in the Yellow Drum (*Nibea Albiflora*) and Identification of Genes Related to Cold Stress. Comp. Biochem. Physiology Part D Genomics Proteomics 28, 80–89. 10.1016/j.cbd.2018.07.003 30005389

[B38] Xu K. Chen D. H. Wang Z. Liu Y. L. Wang Z. Niu B. (2019). Sequencing Analysis and Expression Characteristcs of AFP Gene in *Bombus Terriestris* . J. Shanxi Agric. Univ. Nat. Sci. Ed. 39 (5), 74–81. 10.13842/j.cnki.issn1671-8151.201901030

[B39] Yan W. (2001). GGEbiplot-A Windows Application for Graphical Analysis of Multienvironment Trial Data and Other Types of Two‐Way Data. Agron. J. 93 (5), 1111–1118. 10.2134/agronj2001.9351111x

[B40] Yan W. Holland J. B. (2010). A Heritability-Adjusted GGE Biplot for Test Environment Evaluation. Euphytica 171 (3), 355–369. 10.1007/s10681-009-0030-5

[B41] Yan W. K. Kang M. S. (2003). GGE Biplot Analysis: A Graphical Tool for Breeders, Geneticists, and Agronomists. Boca Raton, Florida: CRC Press.

[B42] Yan W. K. (1999). Methodology of Cultivar Evaluation Based on Yield Trial Data-With Special Reference to Winter Wheat in Ontario. Guelph: University of Guelph.

[B43] Yu W. B. Zhu K. H. Guo H. Y. Zhang N. Sun X. X. Wu N. (2017). Cloning and Expression Analysis of *MHC* II β Gene in *Trachinotus Ovatus* . South China Fish. Sci. 13 (4), 69–79. 10.3969/j.issn.2095-0780.2017.04.009

[B44] Zhang J. F. Tao X. F. Han B. S. (2020). Function, Evolution, and Application of Antifreeze Proteins in Antarctic Fish. J. Fish. Sci. China 27 (3), 355–361. 10.3724/SP.J.1118.2020.19213

[B45] Zhong Q. W. Fan T. J. (2002). Advances in Fish Antifreeze Protein Research. Sheng Wu Hua Xue Yu Sheng Wu Wu Li Xue Bao (Shanghai) 34 (2), 124–130. 10.1007/BF02943277 12007008

