# Supplementary material for: Interpretation of the Genotype by Tissue Interactions of Four Genes (AFP1, CIRP, YB-1, and HMGB1) in Takifugu rubripes Under Different Low-Temperature Conditions
Source: Front Mol Biosci. 2022 Jun 30;9:897935. doi: 10.3389/fmolb.2022.897935 (PMC9280165; doi:10.3389/fmolb.2022.897935)
Supplement: Supplementary file 1 [file DataSheet1.docx]

**Table**

Expression of AFP1, CIRP, YB-1, and HMGB1 in different tissues from fish cultured at different temperatures

(Raw data)

| Water temperature | Tissue | Gene | Repeat group 1 | Repeat group 2 | Repeat group 3 |
| --- | --- | --- | --- | --- | --- |
|  | Brain | AFP1 | 1050.212 | 1018.540 | 1132.146 |
|  | Brain | CIRP | 15.779 | 29.650 | 14.520 |
|  | Brain | HMGB1 | 61.440 | 74.908 | 124.220 |
|  | Brain | YB-1 | 28.725 | 33.862 | 43.544 |
|  | Heart | AFP1 | 6699.010 | 6079.569 | 6101.565 |
|  | Heart | CIRP | 30.064 | 40.785 | 38.319 |
|  | Heart | HMGB1 | 31.828 | 26.375 | 32.153 |
|  | Heart | YB-1 | 137.258 | 169.312 | 236.441 |
|  | Intestine | AFP1 | 1296.313 | 1162.254 | 1387.768 |
|  | Intestine | CIRP | 6.147 | 8.224 | 8.111 |
|  | Intestine | HMGB1 | 3.594 | 3.090 | 3.573 |
|  | Intestine | YB-1 | 15.342 | 19.268 | 23.962 |
|  | Kidney | AFP1 | 2461.160 | 2716.284 | 2680.878 |
|  | Kidney | CIRP | 10.777 | 13.737 | 14.825 |
|  | Kidney | HMGB1 | 6.165 | 7.606 | 7.536 |
|  | Kidney | YB-1 | 13.478 | 14.444 | 21.391 |
|  | Liver | AFP1 | 1498.501 | 1863.959 | 2021.672 |
|  | Liver | CIRP | 8.574 | 9.513 | 22.471 |
|  | Liver | HMGB1 | 17.832 | 18.088 | 19.883 |
|  | Liver | YB-1 | 26.247 | 28.414 | 29.632 |
|  | Muscle | AFP1 | 5050.061 | 5626.871 | 7672.438 |
| 5℃ | Muscle | CIRP | 40.504 | 153.277 | 75.583 |
|  | Muscle | HMGB1 | 66.046 | 61.921 | 71.876 |
|  | Spleen | YB-1 | 2838.165 | 3599.288 | 4106.973 |
|  | Spleen | AFP1 | 3477.550 | 3538.573 | 3521.844 |
|  | Spleen | CIRP | 15.670 | 20.966 | 20.966 |
|  | Spleen | HMGB1 | 14.513 | 14.834 | 17.684 |
|  | Spleen | YB-1 | 26.175 | 29.547 | 31.304 |
|  | Skin | AFP1 | 7726.891 | 6415.825 | 8059.403 |
|  | Skin | CIRP | 15.454 | 15.779 | 14.025 |
|  | Skin | HMGB1 | 11.367 | 11.155 | 12.722 |
|  | Skin | YB-1 | 129.565 | 127.831 | 198.428 |
|  | Gonad | AFP1 | 3163.044 | 2674.845 | 2962.267 |
|  | Gonad | CIRP | 8.754 | 8.693 | 9.986 |
|  | Gonad | HMGB1 | 19.055 | 22.374 | 25.046 |
|  | Gonad | YB-1 | 16.817 | 20.777 | 25.947 |
|  | Brain | AFP1 | 549.118 | 644.025 | 861.659 |
|  | Brain | CIRP | 4.723 | 9.189 | 8.339 |
|  | Brain | HMGB1 | 586.705 | 768.815 | 697.714 |
|  | Brain | YB-1 | 1.733 | 2.209 | 2.287 |
|  | Heart | AFP1 | 1168.931 | 1653.119 | 1676.195 |
|  | Heart | CIRP | 29.650 | 60.968 | 49.866 |
|  | Heart | HMGB1 | 264.384 | 441.568 | 409.151 |
|  | Heart | YB-1 | 23.490 | 29.939 | 26.427 |
|  | Intestine | AFP1 | 962.721 | 1439.123 | 1479.583 |
|  | Intestine | CIRP | 12.125 | 12.906 | 13.361 |
|  | Intestine | HMGB1 | 59.157 | 75.924 | 76.984 |
|  | Intestine | YB-1 | 1.584 | 6.426 | 7.381 |
| 8℃ | Kidney | AFP1 | 1735.306 | 3149.647 | 2938.7250 |
|  | Kidney | CIRP | 5.502 | 8.282 | 9.000 |
|  | Kidney | HMGB1 | 69.382 | 62.098 | 78.058 |
|  | Kidney | YB-1 | 0.086 | 1.226 | 2.384 |
|  | Liver | AFP1 | 446.023 | 523.111 | 498.335 |
|  | Liver | CIRP | 4.377 | 7.012 | 6.498 |
|  | Liver | HMGB1 | 74.361 | 94.124 | 100.182 |
|  | Liver | YB-1 | 3.235 | 6.561 | 6.426 |
|  | Muscle | AFP1 | 1060.828 | 1952.321 | 2420.307 |
|  | Muscle | CIRP | 27.665 | 49.180 | 45.569 |
|  | Muscle | HMGB1 | 124.197 | 115.079 | 125.061 |
|  | Spleen | YB-1 | 322.685 | 569.671 | 636.486 |
|  | Spleen | AFP1 | 3149.647 | 3171.555 | 3215.828 |
|  | Spleen | CIRP | 6.634 | 14.221 | 16.000 |
|  | Spleen | HMGB1 | 76.984 | 117.498 | 108.872 |
|  | Spleen | YB-1 | 0.238 | 0.070 | 0.367 |
|  | Skin | AFP1 | 855.707 | 867.653 | 956.071 |
|  | Skin | CIRP | 17.387 | 18.765 | 18.252 |
|  | Skin | HMGB1 | 65.186 | 54.0601 | 59.157 |
|  | Skin | YB-1 | 50.004 | 76.319 | 72.705 |
|  | Gonad | AFP1 | 342.740 | 413.279 | 592.624 |
|  | Gonad | CIRP | 5.388 | 7.412 | 8.282 |
|  | Gonad | HMGB1 | 40.126 | 30.621 | 35.175 |
|  | Gonad | YB-1 | 1.278 | 1.807 | 2.368 |
|  | Brain | AFP1 | 162.367 | 142.820 | 160.025 |
|  | Brain | CIRP | 14.928 | 25.812 | 15.348 |
|  | Brain | HMGB1 | 97.535 | 103.874 | 111.677 |
|  | Brain | YB-1 | 23.285 | 30.575 | 35.384 |
|  | Heart | AFP1 | 422.354 | 389.913 | 488.250 |
|  | Heart | CIRP | 10.196 | 15.562 | 13.177 |
|  | Heart | HMGB1 | 20.896 | 17.066 | 21.185 |
|  | Heart | YB-1 | 101.109 | 107.688 | 126.818 |
|  | Intestine | AFP1 | 295.423 | 299.604 | 344.377 |
|  | Intestine | CIRP | 7.674 | 7.944 | 8.456 |
|  | Intestine | HMGB1 | 13.219 | 12.932 | 14.691 |
|  | Intestine | YB-1 | 30.669 | 29.718 | 38.005 |
|  | Kidney | AFP1 | 245.430 | 250.729 | 323.471 |
|  | Kidney | CIRP | 21.258 | 19.027 | 18.000 |
|  | Kidney | HMGB1 | 16.994 | 16.688 | 16.253 |
|  | Kidney | YB-1 | 40.867 | 54.179 | 57.197 |
|  | Liver | AFP1 | 281.449 | 458.550 | 357.980 |
| 13℃ | Liver | CIRP | 16.336 | 20.112 | 28.246 |
|  | Liver | HMGB1 | 90.879 | 89.672 | 108.214 |
|  | Liver | YB-1 | 60.448 | 70.304 | 76.044 |
|  | Muscle | AFP1 | 355.673 | 402.765 | 405.460 |
|  | Muscle | CIRP | 15.562 | 18.252 | 16.564 |
|  | Muscle | HMGB1 | 49.542 | 49.551 | 52.353 |
|  | Spleen | YB-1 | 1901.018 | 2057.013 | 2043.93 |
|  | Spleen | AFP1 | 332.457 | 404.507 | 427.317 |
|  | Spleen | CIRP | 4.958 | 5.656 | 5.314 |
|  | Spleen | HMGB1 | 8.422 | 8.955 | 9.907 |
|  | Spleen | YB-1 | 18.520 | 20.014 | 22.826 |
|  | Skin | AFP1 | 504.398 | 421.527 | 398.237 |
|  | Skin | CIRP | 13.737 | 13.547 | 21.258 |
|  | Skin | HMGB1 | 31.745 | 26.232 | 31.354 |
|  | Skin | YB-1 | 62.176 | 61.953 | 76.753 |
|  | Gonad | AFP1 | 4.238 | 3.730 | 4.671 |
|  | Gonad | CIRP | 2.281 | 2.056 | 2.5491 |
|  | Gonad | HMGB1 | 3.331 | 3.010 | 3.487 |
|  | Gonad | YB-1 | 2.651 | 2.665 | 3.224 |
|  | Brain | AFP1 | 35.035 | 36.392 | 29.026 |
|  | Brain | CIRP | 5.656 | 9.382 | 6.868 |
|  | Brain | HMGB1 | 26.328 | 26.863 | 30.938 |
|  | Brain | YB-1 | 13.328 | 14.250 | 14.576 |
|  | Heart | AFP1 | 47.089 | 36.739 | 38.704 |
|  | Heart | CIRP | 10.410 | 14.723 | 12.210 |
|  | Heart | HMGB1 | 15.407 | 15.168 | 17.698 |
|  | Heart | YB-1 | 63.066 | 67.435 | 84.827 |
|  | Intestine | AFP1 | 50.949 | 49.533 | 47.697 |
|  | Intestine | CIRP | 0.806 | 1.042 | 1.189 |
|  | Intestine | HMGB1 | 0.921 | 0.981 | 1.105 |
|  | Intestine | YB-1 | 4.320 | 4.255 | 5.324 |
|  | Kidney | AFP1 | 155.851 | 141.437 | 118.636 |
|  | Kidney | CIRP | 6.773 | 7.310 | 6.276 |
|  | Kidney | HMGB1 | 4.145 | 4.346 | 5.111 |
|  | Kidney | YB-1 | 9.162 | 10.123 | 10.340 |
|  | Liver | AFP1 | 26.049 | 60.930 | 84.649 |
|  | Liver | CIRP | 8.224 | 8.055 | 11.712 |
|  | Liver | HMGB1 | 17.203 | 17.574 | 18.140 |
|  | Liver | YB-1 | 43.451 | 56.835 | 59.157 |
|  | Muscle | AFP1 | 217.583 | 213.229 | 153.047 |
|  | Muscle | CIRP | 16.111 | 15.670 | 15.242 |
| 18℃ | Muscle | HMGB1 | 31.307 | 37.818 | 37.768 |
|  | Spleen | YB-1 | 2336.056 | 2539.327 | 3256.404 |
|  | Spleen | AFP1 | 99.312 | 93.976 | 80.802 |
|  | Spleen | CIRP | 3.837 | 4.084 | 4.027 |
|  | Spleen | HMGB1 | 4.297 | 4.713 | 5.094 |
|  | Spleen | YB-1 | 4.966 | 5.557 | 6.928 |
|  | Skin | AFP1 | 157.892 | 134.783 | 142.472 |
|  | Skin | CIRP | 4.531 | 4.316 | 4.287 |
|  | Skin | HMGB1 | 13.663 | 10.268 | 15.424 |
|  | Skin | YB-1 | 70.782 | 73.557 | 81.504 |
|  | Gonad | AFP1 | 1.340 | 0.780 | 0.956 |
|  | Gonad | CIRP | 2.549 | 2.620 | 3.031 |
|  | Gonad | HMGB1 | 3.807 | 2.941 | 4.121 |
|  | Gonad | YB-1 | 1.039 | 0.874 | 1.099 |
|  | Brain | AFP1 | 35.035 | 36.392 | 29.026 |
|  | Brain | CIRP | 5.656 | 9.382 | 6.868 |
|  | Brain | HMGB1 | 26.328 | 26.863 | 30.938 |
|  | Brain | YB-1 | 13.328 | 14.250 | 14.576 |
